# MiR-185-5p targets RAB35 gene to regulate tumor cell-derived exosomes-mediated proliferation, migration and invasion of non-small cell lung cancer cells

**DOI:** 10.18632/aging.203483

**Published:** 2021-09-09

**Authors:** Hongqing Wen, Zhiyan Liu, Jingjing Tang, Lina Bu

**Affiliations:** 1 Department of Respiratory and Critical Care Medicine, Xi'an No. 3 Hospital, The Affiliated Hospital of Northwest University, Xi’an 710018, Shaanxi, P.R. China

**Keywords:** non-small cell lung cancer (NSCLC), miR-185-5p, RAB35, exosomes

## Abstract

Introduction: Non-small cell lung cancer (NSCLC) is the most common malignant tumor, and its recurrence and metastasis are the main causes of death. Recently, there is evidence that tumor derived exosomes play an important role in the occurrence and development of NSCLC.

Objective’s methods: First, the expression of miR-185-5p and RAB35 in NSCLC tissues, paracancerous tissues, NSCLC cell lines and normal human bronchial epithelial cell line was detected. Then, a series of gain-and loss-of-function assays were performed to validate the effects of miR-185-5p or RAB35 effects on A549 and H2170 cells proliferation, migration and invasion. Next, online bioinformatics analysis and luciferase reporter were used to predict and validate the targeting relationship of miR-185-5p and RAB35. Finally, tumor cell-derived exosomes with genetic downregulation of RAB35 or overexpression of miR-185-5p were co cultured with their parental cells to verify the regulatory role of RAB35 on exosome secretion and function.

Results: In NSCLC tissues and cell lines, miR-185-5p was downregulated, while RAB35 was significantly upregulated. Overexpression of miR-185-5p or knockdown of RAB35 expression inhibited cell proliferation, migration and invasion. Furthermore, we elucidated that RAB35 is a direct target of miR-185-5p. Additionally, exosomes derived from tumor cells restored cell proliferation, migration and invasion, whereas exosomes secreted by tumor cells with downregulation of RAB35 expression or overexpression of miR-185-5p lost their ability to restore cell proliferation, migration and invasion.

Conclusions: Our results demonstrate that miR-185-5p inhibits tumor cell-derived exosomes-mediated proliferation, migration and invasion of NSCLC cells by downregulating RAB35 expression.

## INTRODUCTION

Lung cancer is a highly prevalent malignancy of the respiratory system, which can be divided into non-small cell lung cancer (NSCLC) and small cell lung cancer (SCLC). NSCLC accounts for 80% - 85% of all lung cancers, with lung adenocarcinoma, squamous cell carcinoma, and large cell carcinoma being the most common [1]. There is a study showing that in renal cell carcinoma progression, miR-126 attenuates the ability of renal cell carcinoma cells to invade and metastasize by targeted inhibition of Rock1 expression [2]. Pan et al. reported that miR-370 was able to inhibit hepatocellular carcinoma progression, which was mediated by targeted inhibition of the expression of PIM1 [3]. Similarly, Yang et al. collected tumor tissues from glioma patients and found that miR-155 was highly expressed by RT qPCR assay, and mechanistic studies showed that miR-155 could promote tumor cell growth by inhibiting caudal type homeobox 1 (CXD-1) [4]. Recently, emerging evidence has demonstrated that miR-185-5p is expressed at low levels in NSCLC cells and the growth of tumor cells is inhibited when miR-185-5p is overexpressed [5].

RAB GTPases belong to one of the important members of the Ras superfamily of small GTPase proteins, and RAB GTPases are expressed in almost all cells [6]. RAB GTPases have been found to be closely associated with transmembrane transport in cells, where they can form active membrane structures and associate with corresponding proteins, affecting extracellular vesicle formation, transport, and fusion [7]. It has been reported that the expression of rab27b, which is the main component of vesicles in exosomes, is significantly correlated with histological types of lung cancer patients, and lung cancer patients with high rab27b expression have a poor prognosis [8]. Hsu Chieh et al. reported that loss of RAB35 function aggravates the accumulation of intracellular vesicles in oligodendrocytes and decreases the number of extracellular vesicles, suggesting that Rab35 plays a direct role in exosome synthesis and secretion [9].

Exosomes are a type of membranous vesicles secreted by cells into the extracellular matrix, which are approximately 30-100 nm in diameter and are usually released outside the cell upon fusion of intracellular vesicles with the cell membrane. Studies have found that exosomes can be secreted by almost all types of cells including tumor cells [10]. Currently, researchers have identified exosomes in plasma, CSF, urine, breast milk, and saliva. Emerging evidence suggests that exosomes secreted by tumor cells into the extracellular space are able to influence the tumor microenvironment and regulate tumor cell proliferation, invasion, migration, and angiogenesis, which in turn inhibit or promote tumor progression [11, 12].

Accumulating evidence suggests that the interaction between miRNAs and Rabs may serve as a new entry point for tumor therapy. Tang et al. showed that miR-720 promoted HeLa cell migration by downregulating RAB35 [13]. In lung cancer, miR-296-3p was reported to be involved in A549 cell growth and metastasis by regulating RabL3 expression [14]. However, the specific mechanism of whether miR-185-5p promotes or inhibits the progression of NSCLC by regulating RAB35 expression to affect the synthesis and secretion of exosomes by NSCLC cells remains unclear. Therefore, the present study will further define the specific mechanism by which miR-185-5p targets the RAB35 gene to affect the development and progression of NSCLC cell-derived exosome mediated tumorigenesis, and this study may contribute to the development of potential treatments for NSCLC.

## RESULTS

### MiR-185-5p inhibited NSCLC cell growth

**Figure 1:**
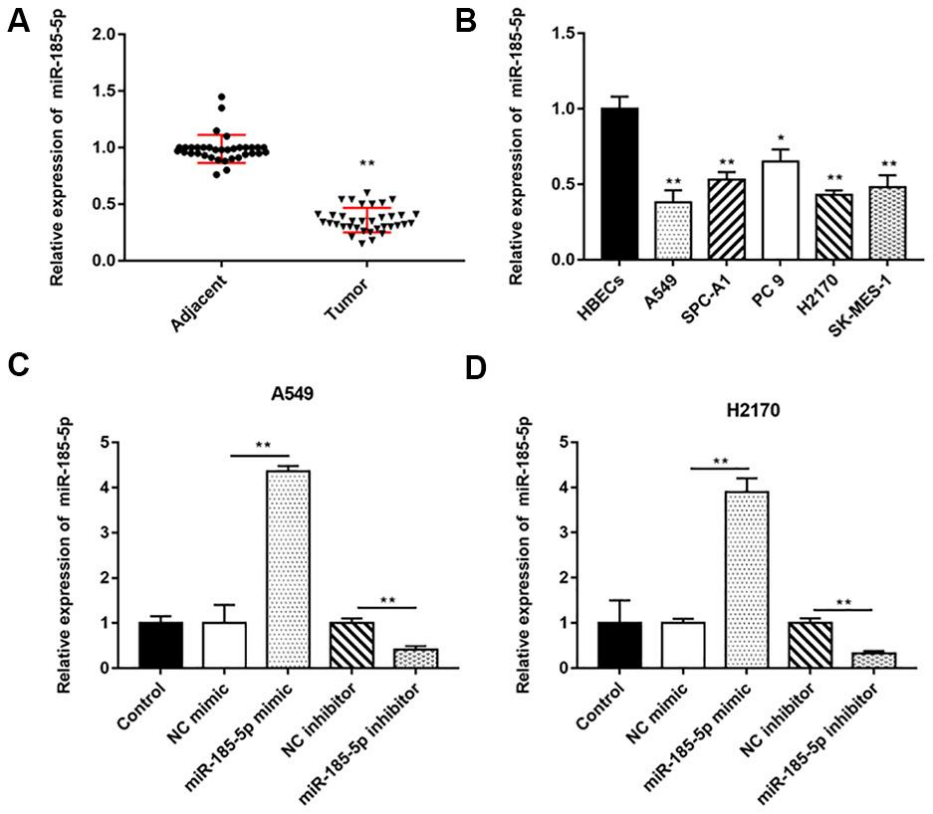
**MiR-185-5p is downregulated in NSCLC tissues and cell lines.** The NSCLC tissues and tissues larger than 5 cm around the tumor were collected (N=70, 35 tumor tissues and 35 adjacent tissues). (**A**) RT-qPCR was used to detect the relative expression of miR-185-5p in tumor tissues and adjacent tissues, and the results showed that miR-185-5p was downregulated in NSCLC tissues. (**B**) Relative expression of miR-185-5p in NSCLC cell lines (A549, SPC-A1, PC 9, H2170 and SK-MES-1 cells) and normal bronchial epithelial cell line (HBECs) were detected by RT-qPCR assay, and the results showed that miR-185-5p was downregulated in NSCLC cell lines. The miR-185-5p mimic, miR-185-5p inhibitor and their negative control were transfected into A549 cells and H2170 cells, respectively. (**C**, **D**) Relative expression of miR-185-5p in A549 cells and H2170 cells was analyzed by RT-qPCR. N=6, * *P*<0.05, ** *P*<0.01.

We extracted total RNA and performed RT-qPCR on 35 pairs of NSCLC tumor tissues and adjacent tissues, and the results indicated that miR-185-5p expression was decreased in lung cancer tissues compared with adjacent tissues (Figure 1A). Similarly, miR-185-5p expression was decreased in NSCLC cells (A549, SPC-A1, PC 9, H2170 and SK-MES-1) compared with HBECs (Figure 1B).

**Figure 2:**
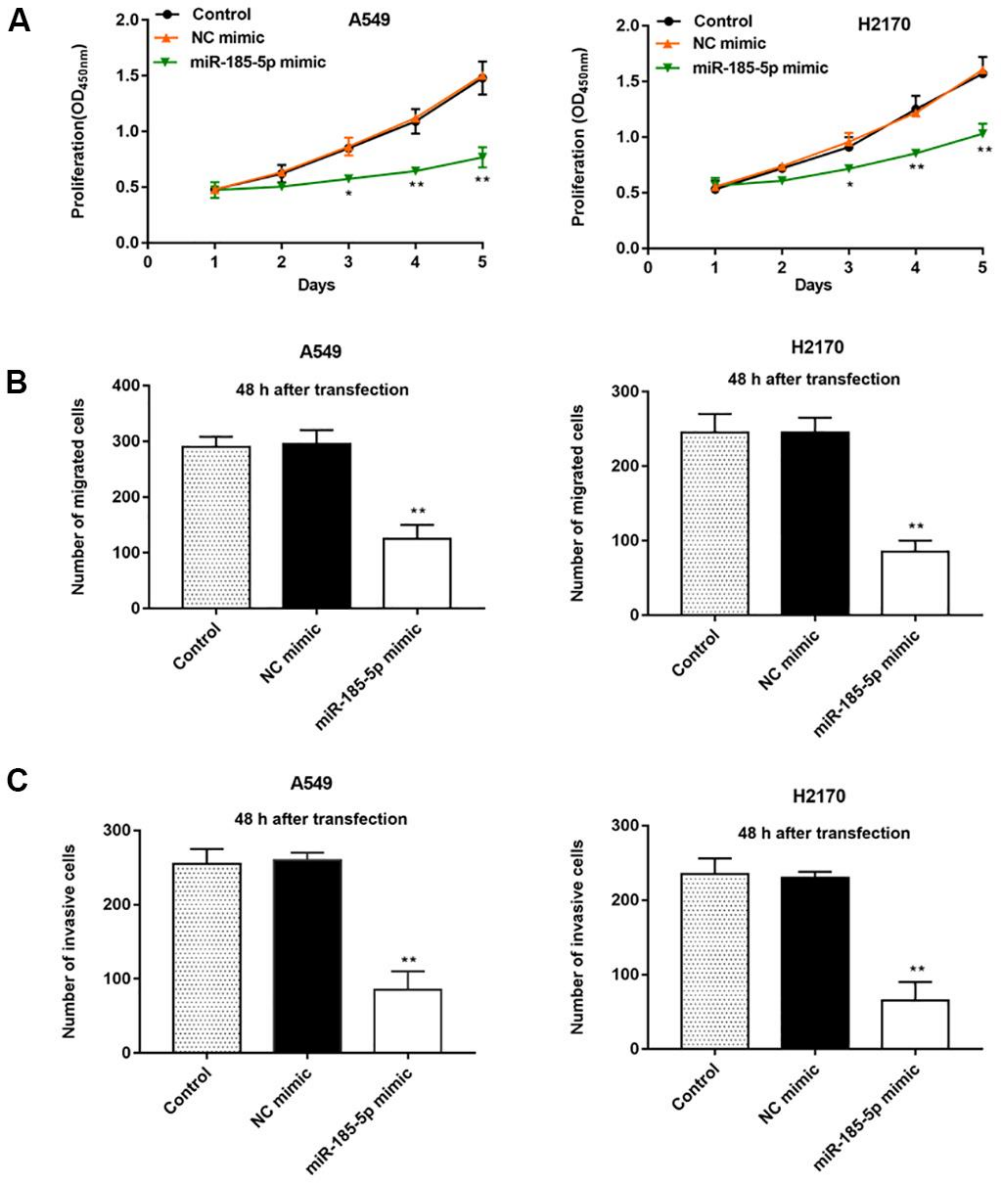
**Overexpression of miR-185-5p inhibits proliferation, migration and invasion of NSCLC cells.** The miR-185-5p mimic and NC mimic were transfected into A549 cells and H2170 cells, respectively. (**A**) The proliferation of A549 and H2170 cells was assessed by CCK-8 assay, and the results showed that miR-185-5p mimic inhibited cell proliferation. (**B**) The migration of A549 and H2170 cells was detected by Transwell cell migration assay, and the results showed that miR-185-5p mimic inhibited cell migration. (**C**) The invasion of A549 and H2170 cells was analyzed by Transwell cell invasion assay, and the results showed that miR-185-5p mimic inhibited cell invasion. N=6, * *P*<0.05, ** *P*<0.01.

Our previous results showed that miR-185-5p was down regulated more fold in A549 and H2170 cells, therefore, we will deeply explore the regulatory role of miR-185-5p in tumor progression in these two types of cells. Then, A549 and H2170 cells were transfected with miR-185-5p mimic and the transfection efficiency was shown in Figure 1C, 1D. Furthermore, we observed that the proliferation ability of A549 and H2170 cells was attenuated upon transfection of miR-185-5p mimics (Figure 2A). Transfection of miR-185-5p mimic inhibited the migration and invasion of A549 and H2170 cells compared with the control (Figure 2B, 2C).

### RAB35 siRNA inhibited NSCLC cell growth

**Figure 3:**
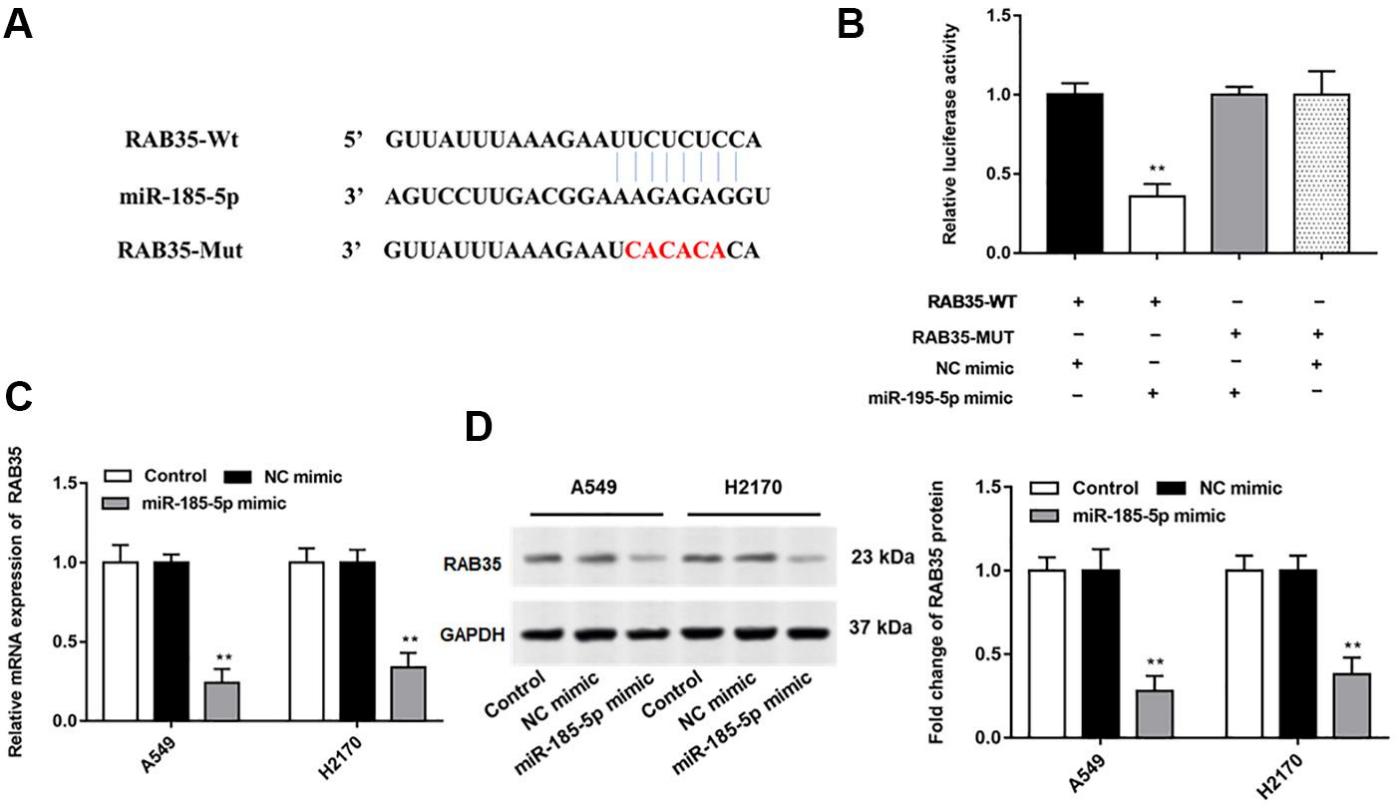
**MiR-185-5p directly targets 3'UTR of RAB35.** (**A**) StarBase 3.0 (http://starbase.sysu.edu.cn/) was used to predict the target genes of miR-185-5p, and we found that miR-185-5p binds to RAB35 mRNA 3'UTR. (**B**) The relative luciferase activity was tested with wild-type and mutant-type RAB35, respectively. (**C**, **D**) The miR-185-5p mimic and NC mimic were transfected into A549 cells and H2170 cells, respectively. The mRNA and protein expression of RAB35 in A549 and H2170 cells was analyzed by RT-qPCR and Western blotting, and the results showed that miR-185-5p mimic inhibited mRNA and protein expression of RAB35. β-actin was used as an invariant internal control for calculating protein-fold changes. N=6, ** *P*<0.01.

Bioinformatics databases (http://starbase.sysu.edu.cn/) were used to predict the potential target genes of miR-185-5p, and RAB35 was screened. We observed no binding relationship between mutated RAN35 and miR-185-5p (Figure 3A). Next, we found that miR-185-5p mimic reduced the luciferase activity of wild-type Rab35 but not mutant Rab35 (Figure 3B). Furthermore, the mRNA and protein expression levels of RAB35 in A549 and H2170 cells were significantly suppressed after transfection with miR-185-5p mimic (Figure 3C, 3D).

**Figure 4:**
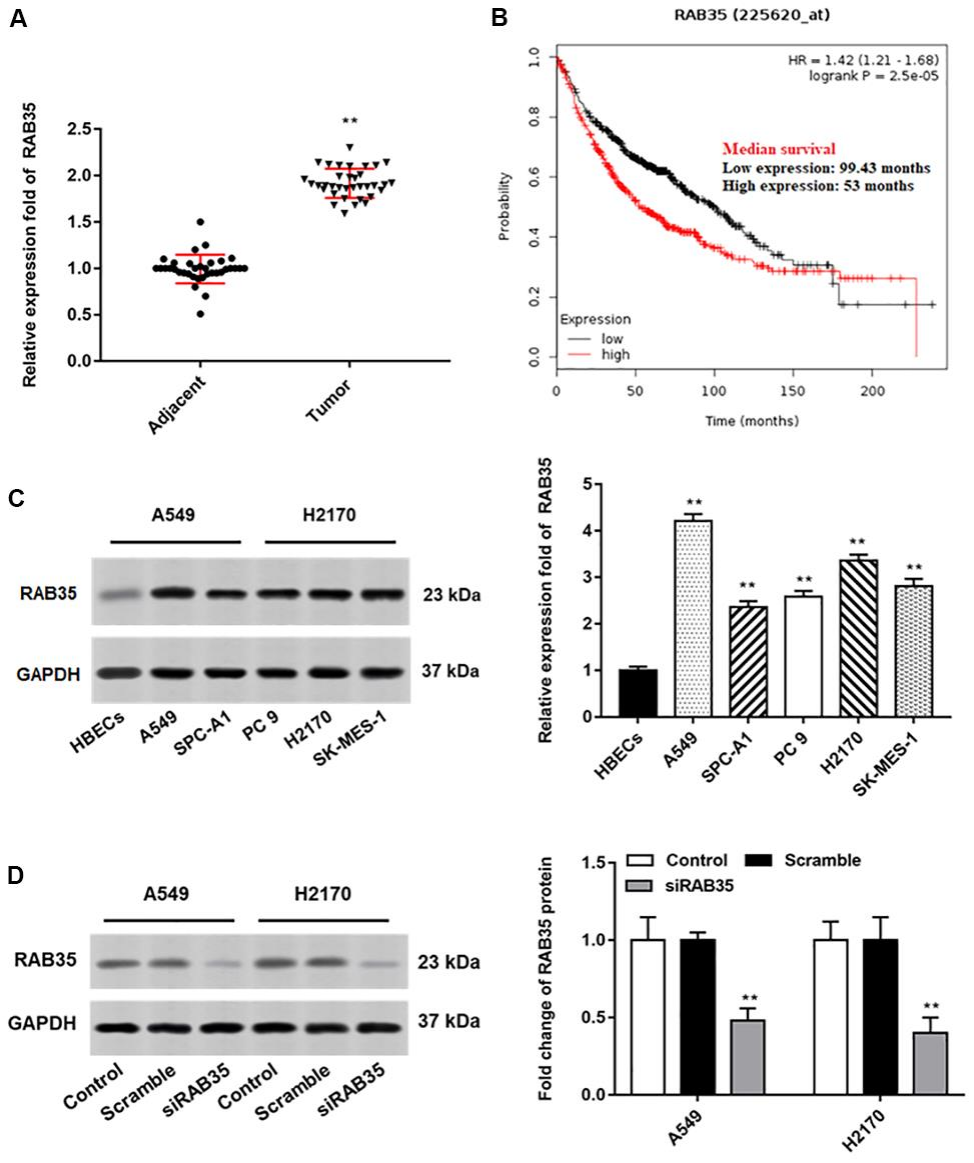
**RAB35 is upregulated in NSCLC tissues and cell lines.** The NSCLC tissues and tissues larger than 5 cm around the tumor were collected (N=70, 35 tumor tissues and 35 adjacent tissues). (**A**) RT-qPCR was used to detect the relative expression of RAB35 in tumor tissues and adjacent healthy tissues, and the results showed that RAB35 was upregulated in NSCLC tissues. (**B**) KM plotter software (http://kmplot.com/analysis/) was used to analyze the expression and survival of RAB35 gene in 1928 lung cancer patients. (**C**) Western blotting was used to detect the relative protein expression of RAB35 in NSCLC cell lines (A549, SPC-A1, PC 9, H2170 and SK-MES-1 cells) and normal bronchial epithelial cell line (HBECs), and the results showed that RAB35 was upregulated in NSCLC cell lines. (**D**) RAB35 siRNA and negative control (Scramble) were transfected into A549 cells and H2170 cells, respectively. The protein expression of RAB35 was analyzed by Western botting. β-actin was used as an internal reference. N=6, ** *P*<0.01.

**Figure 5:**
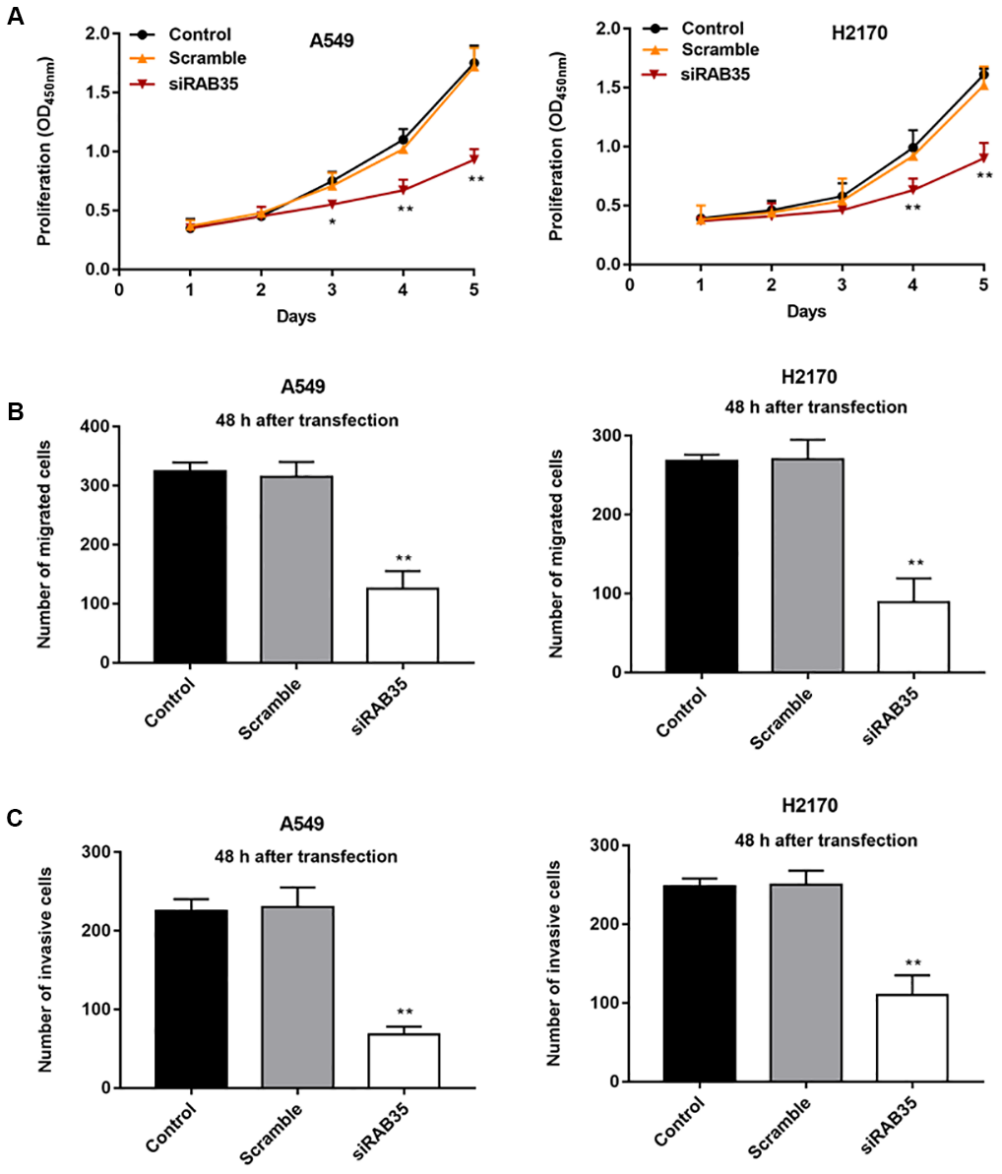
**Knockdown of RAB35 inhibits proliferation, migration and invasion of NSCLC cells.** RAB35 siRNA and negative control (Scramble) were transfected into A549 cells and H2170 cells, respectively. (**A**) CCK-8 assay was used to measure the proliferation of A549 and H2170 cells at day 1, 2, 3, 4, 5 after transfection, and the results showed that RAB35 knockdown inhibited cell proliferation. (**B**) The migration of A549 and H2170 cells was detected by Transwell cell migration assay, and the results showed that RAB35 knockdown inhibited cell migration. (**C**) The invasion of A549 and H2170 cells was analyzed by Transwell cell invasion assay, and the results showed that RAB35 knockdown inhibited cell invasion. β-actin was used as the loading control. N=6, ** *P*<0.01.

Then, we found that the expression of RAB35 was higher in tumor tissues than in adjacent tissues (Figure 4A). Moreover, biological database (http://kmplot.com/analysis/) exhibited the relationship between RAB35 expression and survival cycle in 1928 lung cancer patients, indicating that the median survival of patients with RAB35 low expression was 99.43 months, while that of patients with RAB35 high expression was only 53 months (Figure 4B). In different NSCLC cell lines, we similarly observed high levels of RAB35 expression (Figure 4C). Next, A549 and H2170 cells were transfected with RAB35 siRNA and the transfection efficiency is shown in Figure 4D. The proliferation ability of A549 and H2170 cells was attenuated upon transfection of RAB35 siRNA (Figure 5A). Transfection of RAB35 siRNA inhibited the migration and invasion of A549 and H2170 cells compared with the control (Figure 5B, 5C).

### MiR-185-5p inhibited NSCLC cell growth by targeting RAB35

**Figure 6:**
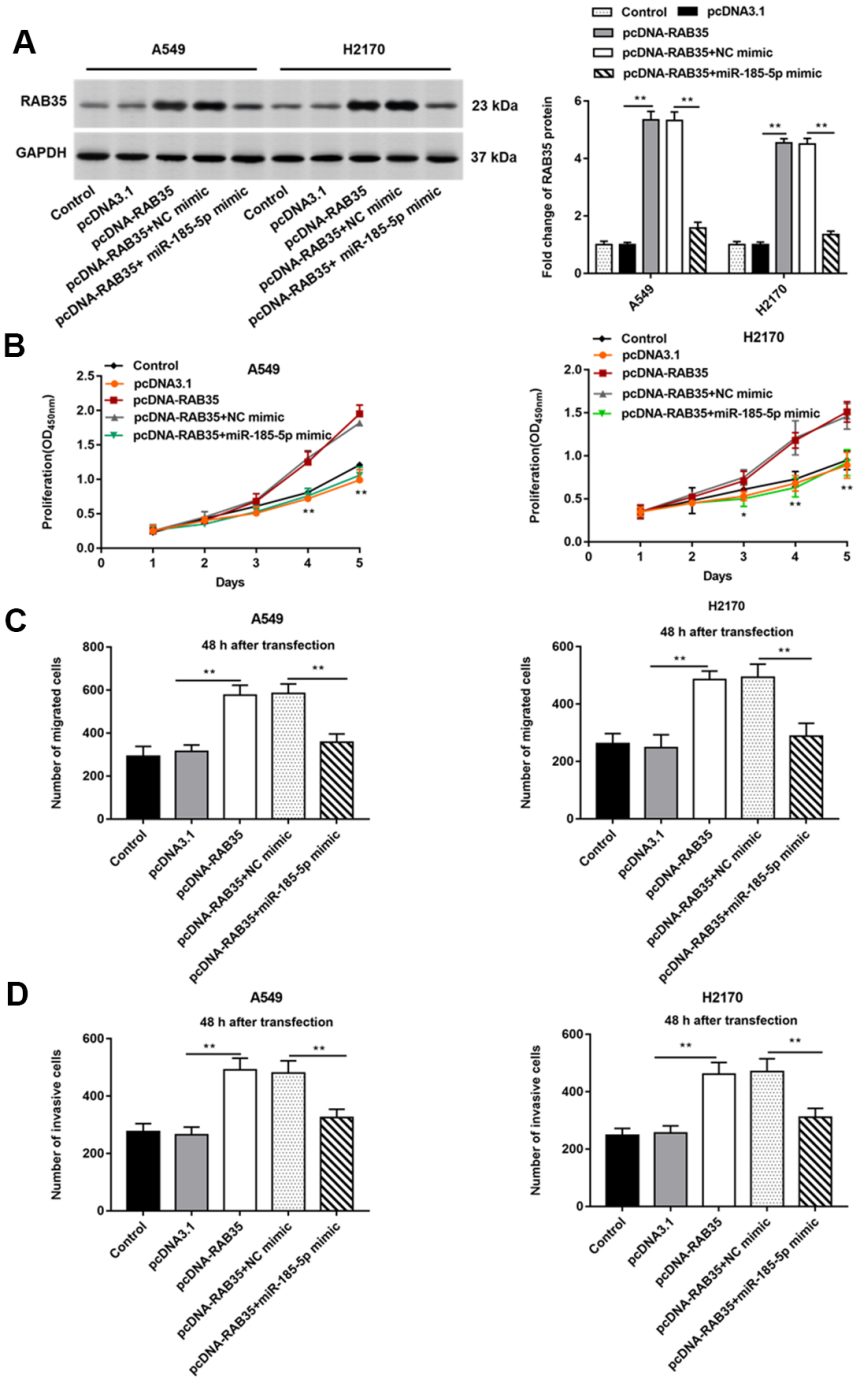
**MiR-185-5p inhibits proliferation, migration and invasion of NSCLC cells by targeting RAB35.** The pcDNA-RAB35 was transfected into A549 cells and H2170 cells alone or together with miR-185-5p mimic, respectively. (**A**) Fold change of RAB35 protein was analyzed by Western blotting, and we found that miR-185-5p mimic reversed the promoting effect of pcDNA-RAB35 on RAB35 protein expression. (**B**) The cell proliferation of A549 and H2170 cells at day 1, 2, 3, 4, 5 after transfection was measure by CCK-8 assay. (**C**) Transwell cell migration assay was used to measure the migration of A549 cells and H2170 cells. (**D**) The cell invasion was detected by Transwell cell invasion assay. We found that miR-185-5p mimic reversed the promoting effect of pcDNA-RAB35 on cell proliferation, migration and invasion. β-actin was used as an internal reference. N=6, ** *P*<0.01.

The A549 and H2170 cells were transfected with pcDNA-RAB35 alone or together with miR-185-5p mimic. We observed that pcDNA-RAB35 promoted RAB35 protein expression, and miR-185-5p mimic inhibited RAB35 protein expression (Figure 6A). Moreover, the pcDNA-RAB35 promoted cell proliferation in A549 and H2170 cells. However, the enhanced cell proliferation by pcDNA-RAB35 was reversed by miR-185-5p mimic (Figure 6B). The ability of migration and invasion of A549 and H2170 cells was enhanced by pcDNA-RAB35, but this change was partially abolished by miR-185-5p mimic (Figure 6C, 6D).

### Effect of RAB35 on exosome secretion from A549 cells

**Figure 7:**
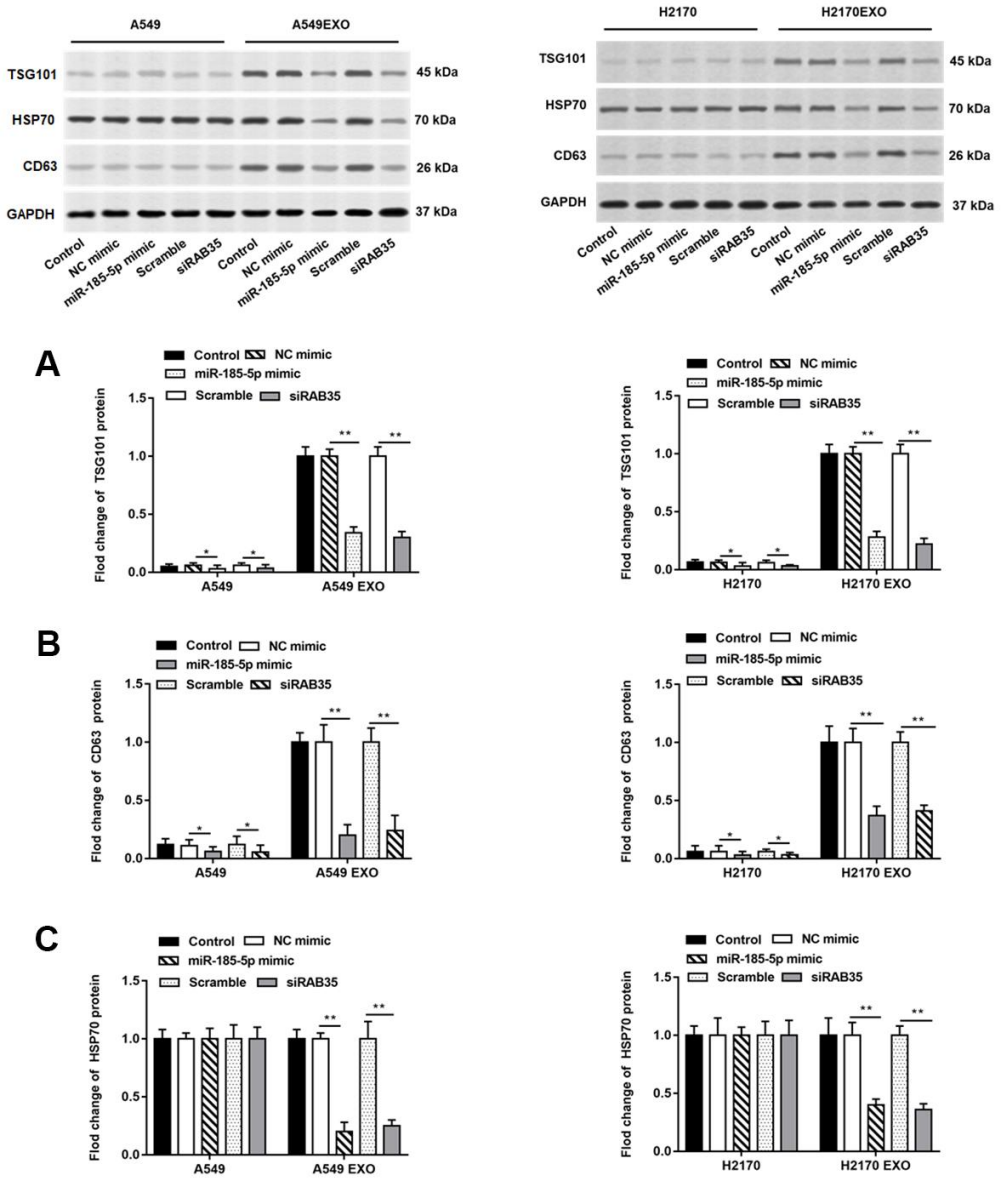
**Knockdown of RAB35 reduces the number of exosomes from tumor cells and inhibits its function.** The miR-185-5p mimic, RAB35 siRNA and their negative control were transfected into A549 and H2170 cells, respectively. Next, the cell culture medium was collected after 48 hours and extracted exosomes. (**A**–**C**) Western blotting was used to detect the relative protein expression of TSG101, CD63 and HSP70 in A549 cells, H2170 cells and their secreted exosomes, and the results suggested that exosomes isolated from A549 and H2170 cells transfected with miR-185-5p mimic and siRAB35 had lower protein expression of TSG101, CD63 and HSP70. β-actin was used as an invariant internal control for calculating protein-fold changes. N=6, ** *P*<0.01.

There have been numerous studies confirming that exosomes secreted by tumor cells promote or inhibit tumor progression by regulating tumor cell growth [15, 16]. It is well known that some proteins including CD63, HSP70 and TSG101 are markers for detecting exosomes. A549 and H2170 cells were transfected with miR-185-5p mimic and RAB35 siRNA for 48 h, and cell culture medium was collected and exosomes were extracted. We found that TSG101 and CD63 proteins were enriched in exosomes secreted by A549 and H2170 cells but not in A549 and H2170 cells. When overexpressing miR-185-5p or interfering with RAB35, we observed suppressed TSG101 protein and CD63 protein expression in tumor cells and their derived exosomes (Figure 7A, 7B). Moreover, HSP70 protein expression was reduced in exosomes secreted by tumor cells transfected with miR-185-5p mimic or RAB35 siRNA (Figure 7C).

### Exosomes secreted by tumor cells with RAB35 knockdown inhibited NSCLC proliferation

**Figure 8:**
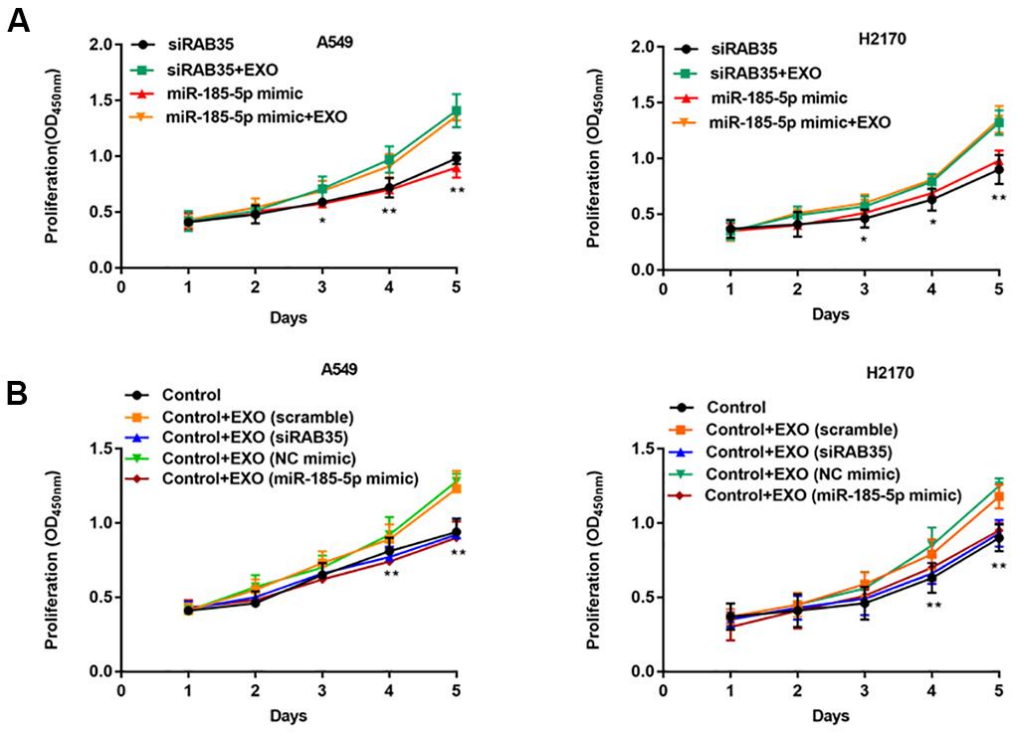
**NSCLC cell-derived exosomes with RAB35 knockdown or miR-185-5p overexpression inhibit NSCLC cell proliferation.** (**A**) A549 cells (H2170 cells) with knockdown of RAB35 or overexpression of miR-185-5p were incubated with exosomes secreted by A549 cells (H2170 cells). CCK-8 assay was used to measure the proliferation of A549 and H2170 cells at day 1, 2, 3, 4, 5 after transfection. (**B**) RAB35 siRNA was transfected as negative control group and co-cultured with the exosomes secreted by RAB35 downregulated or miR-185-5p overexpression A549 cells and H2170 cells, respectively. The cell proliferation was measure by CCK-8 assay. N=6, ** *P*<0.01.

Exosomes secreted by A549 and H2170 cells were incubated with A549 and H2170 cells with knockdown of RAB35 expression. We observed that exogenous exosomes restored the proliferation ability of A549 and H2170 cells with knockdown of RAB35 expression (Figure 8A). Further, exosomes secreted by normal A549 cells (transfected with scramble or NC mimic) restored cell proliferation ability compared with A549 cells with knockdown of RAB35 expression (transfected with siRAB35 or miR-185-5p mimic), whereas exosomes secreted by A549 cells with knockdown of RAB35 expression (transfected with siRAB35 or miR-185-5p mimic) again inhibited cell proliferation (Figure 8B). The same results were validated in H2170 cells.

### Exosomes secreted by tumor cells with RAB35 knockdown inhibited NSCLC migration and invasion

**Figure 9:**
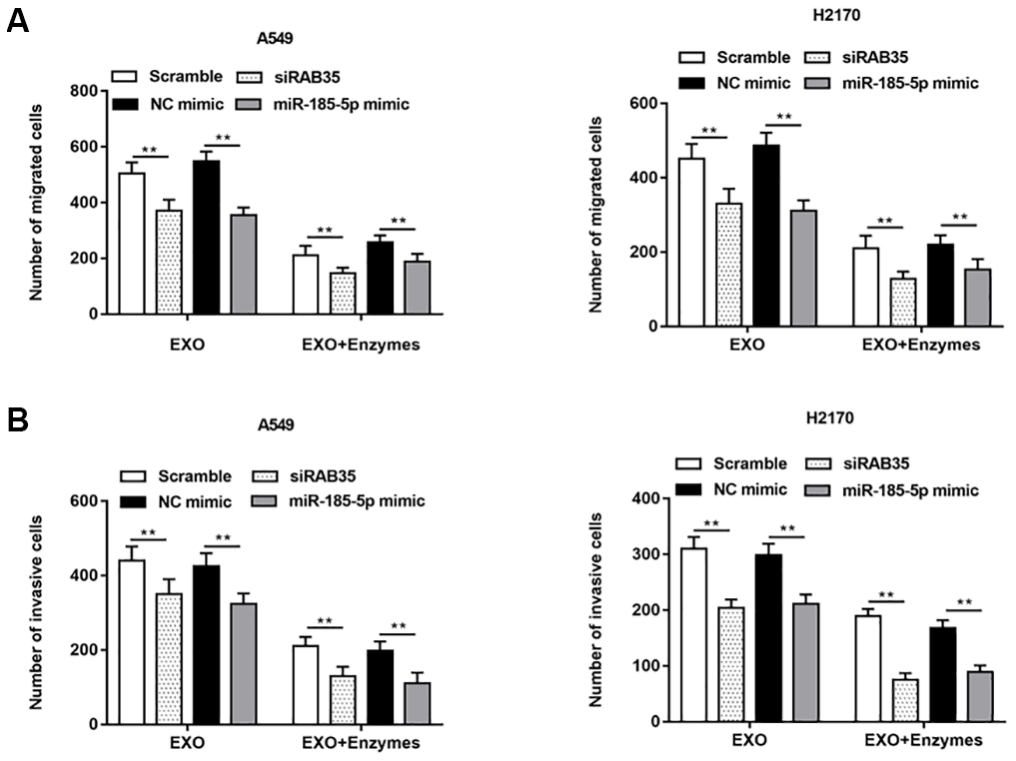
**NSCLC cell-derived exosomes with RAB35 knockdown or miR-185-5p overexpression (Enzyme treated) inhibited NSCLC cell migration and invasion.** The exosomes secreted by A549 cells and H2170 cells were treated with enzyme and then added into the two kinds of cell models with downregulation of RAB35. (**A**) Transwell cell migration assay was used to measure the migration of A549 cells and H2170 cells, and the results showed that the promoting effects of exosomes secreted by A549 and H2170 cells on cell migration and invasion were attenuated under the action of enzymes. (**B**) The cell invasion was detected by Transwell cell invasion assay. N=6, ** *P*<0.01.

Exosomes secreted by A549 cells were treated with enzymes, and then the enzyme treated exosomes were co incubated with A549 cells with knockdown of RAB35 expression. We observed that cell migration (Figure 9A) and invasion (Figure 9B) were inhibited. The same results were validated in H2170 cells.

### Diminished ability of exosomes derived from A549 cells overexpressing miR-185-5p to induce tumorigenesis in nude mice

**Figure 10:**
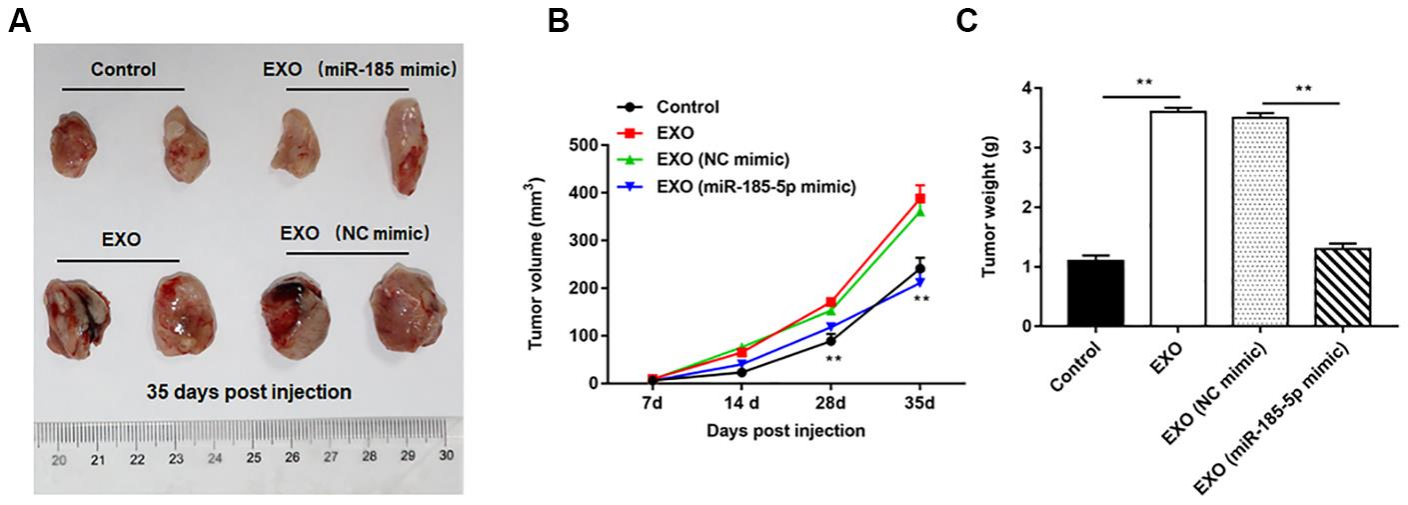
**Exosomes from A549 cells overexpressing miR-185-5p inhibit NSCLC progression *in vivo*.** Sixty-four nude mice were intraperitoneally injected with A549 cells, and then randomly divided into control group (normal saline), EXO group (A549 cell-derived exosomes), NC mimic group (exosomes derived from A549 cells transfected with NC mimic) and miR-185-5p mimic group (exosomes derived from A549 cells transfected with miR-185-5p mimic), with 4 nude mice in each group by intraperitoneal injection. On the days 7, 14, 28 and 35 after injection, three mice were sacrificed in each group, and tumor tissues were taken to determine the tumor volume and weight. (**A**) Representative tumor images at day 35 post injection. (**B**) Tumor volume. (**C**) Tumor weight. We found that injection of exosomes derived from A549 cells transfected with miR-185-5p mimic reduced tumor weight and volume in nude mice. N=4 for each group. **p< 0.01.

Nude mice tumor bearing models were constructed by intraperitoneal injection of A549 cells, and the normal saline, A549 cell-derived exosomes, A549 cell (transfected with NC mimic)-derived exosomes and A549 cell (transfected with miR-185-5p mimic)-derived exosomes were injected into nude mice, respectively. We observed that A549 cell-derived exosomes induced tumorigenesis more readily than A549 cells, as indicated by the larger volume and weight of the tumors formed in nude mice, and the representative tumor images are shown in Figure 10A. Furthermore, the volume (Figure 10B) and weight (Figure 10C) of tumors formed by exosome treated nude mice derived from 549 cells overexpressing miR-185-5p were significantly reduced compared to those from exosome treated nude mice derived from normal A549 cells (transfected with NC mimic).

## DISCUSSION

Studies have shown that miR-185-5p is able to regulate the growth of a variety of tumor cells, and it affects the prognosis of patients. The investigators found that miR-185-5p attenuated the proliferative capacity of tumor cells, promoted tumor cell apoptosis, and delayed prostate cancer progression by downregulating RNCR3 expression [17]. Yu et al. reported that miR-185-5p was under-expressed in lung squamous cell carcinoma tumor tissues and NSCLC cell lines, and low miR-185-5p expression was an independent risk factor for poor patient prognosis [18]. Additional studies reported that LncRNA MALAT1 and MDM4 were upregulated in NSCLC cells. MiR-185-5p, a MALAT1 target, can directly target MDM4 and significantly inhibit NSCLC cell growth when overexpressed [5]. Pei et al. reported that miR-185-5p was highly expressed in normal A549 cells compared with cisplatin sensitive A549 cells, and the sensitivity of A549 cells to cisplatin was increased when miR-185-5p mimic were transfected, while miR-185-5p inhibitor promoted cisplatin resistance in A549 cells [19]. In the present study, we found that miR-185-5p was downregulated in NSCLC tissues and cell lines, and overexpression of miR-185-5p inhibited the proliferation, migration, and invasion of NSCLC cells. Moreover, RAB35 was identified as a target gene of miR-185-5p, and RAB35 overexpression plasmid reversed the regulatory effect of miR-185-5p on NSCLC cells.

Numerous studies have proved that the high expression of RAB35 was related to the malignancy degree and poor prognosis of tumors. Zhu et al. found that RAB35 expression increased in MCF-7 breast cancer cells, and significantly inhibited Wnt5a-induced cell proliferation after knocking down RAB35 expression A study showed that RAB35 was abnormally highly expressed in breast cancer cells MCF-7, and when RAB35 expression was inhibited, Wnt5a induced cell proliferation was also inhibited [20]. Duan et al. reported that upon silencing of RAB35, the stability of GIT2 was disrupted, and the level of phosphorylation was reduced, further inhibiting the polarization and migration of NSCLC cells [21]. In addition, RAB35 was identified as a binding protein of lncRNA HOTAIR and its expression was positively regulated by RNA HOTAIR, accelerating the metastasis of hepatocellular carcinoma cancer cells [22]. In a study related to leukemia, investigators found that RAB35 has a strong promoting effect on cancer cell invasion, metastasis and immune escape [23]. The above reports further corroborate our findings and suggest that RAB35 plays an important role in NSCLC progression.

Exosomes are important components that constitute the tumor microenvironment and can participate in angiogenesis to accelerate tumor progression through intercellular information communication [24, 25]. For example, mesenchymal stem cells undergo morphological and functional alterations under the stimulation of exosomes secreted by colorectal cancer cells, and this change will further affect tumor cell growth and metastasis [26]. Wu et al. suggested that the NF-κB pathway in macrophages was rapidly activated upon stimulation by exosomes secreted by gastric cancer cells, accelerating tumor metastasis [27]. Another study found that exosomes were able to mediate membrane transport of metastasis associated protein 1 (MTA1) in breast cancer cells, which had a positive regulatory effect on hypoxia and estrogen signaling [28]. As an important part of tumor microenvironment, exosomes play an important role in the process of drug resistance. Similarly, Qu et al. reported that sorafenib induced apoptosis was abrogated under exosome intervention, resulting in decreased drug sensitivity of hepatocellular carcinoma cells, leading to poor prognosis of liver cancer patients [29]. In this study, we found that the synthesis and secretion of exosomes were inhibited in A549 and H2170 cells after transfection with siRAB35 or miR-185-5p mimic. Furthermore, we found that exosomes secreted by A549 and H2170 cells were able to restore the growth of parental cells, suggesting that exosomes play an important role in NSCLC progression.

In this study, a nude mouse tumor bearing model was constructed by intraperitoneal injection of A549 cells to investigate the effects of A549 cell-derived exosomes on tumor progression. In fact, we first consider to establish the lung orthotopic xenograft tumor nude mice model, unfortunately, the results are unsatisfactory, which is also a pitfall of *in vivo* studies. Later we will construct suitable animal models to simulate the tumor microenvironment in the lung, and further verify the conclusions of this study. In addition, A549 cells and H2170 cells were selected for subsequent experiments in this study, and we will next investigate them in more NSCLC cell lines. Similarly, to further demonstrate the process of exosome secretion and exosome uptake by NSCLC cells, we will consider the identification of exosomes by nanoparticle tracking analysis (NTA).

Taken together, as a target of miR-185-5p, RAB35 promotes the synthesis and secretion of exosomes to mediate NSCLC cell growth *in vitro* and metastasis *in vivo*, and our findings may become a potential idea for the treatment of NSCLC.

## MATERIALS AND METHODS

### Animals

Adult nude mice (20-25 g) were obtained from Henan Experimental Animal Center (Henan, China). All mice were adaptively housed for one week with free access to food and water. Sixty-four nude mice were intraperitoneally injected with A549 cells, and then divided into three groups: control group (normal saline), EXO group (A549 cell-derived exosomes), NC mimic group (exosomes derived from A549 cells transfected with NC mimic) and miR-185-5p mimic group (exosomes derived from A549 cells transfected with miR-185-5p mimic), with 4 nude mice in each group by intraperitoneal injection. On the days 7, 14, 28 and 35 after injection, three mice randomly selected in each of the four groups were euthanized, and tumor tissues were collected for subsequent studies. All animal experimental procedures were approved by the ethics committee of Xi’an No. 3 Hospital, the Affiliated Hospital of Northwest University (Xi’an, China).

### Clinical specimens

Tumor tissues and adjacent tissues from 35 NSCLC patients were collected with informed consent from each subject. The age distribution of the patients was (46.2 ± 8.1) years. This study was approved by the ethics committee of Xi’an No. 3 Hospital, the Affiliated Hospital of Northwest University (Xi’an, China).

### Cell transfection

The human NSCLC cell lines were obtained from ATCC (Manassas, VA, USA). The cells were cultured in RPMI 1640 medium (Gibco, Rockville, MD) containing 10 % FBS and 100 U/ml penicillin and 100 μg/mL streptomycin (Sigma, St. Louis, MO, USA) with 5% CO_2_ at 37° C. MiR-185-5p mimic, and pcDNA-RAB35 and their negative control were performed from RiboBio Co., Ltd (Guangzhou, China) and transfected by using RiboBio Transfection Kit (RiboBio Co., Ltd). RAB35 siRNA and Scramble were performed from Santa Cruz Biotechnology (Santa Cruz, CA, USA) and using Lipofectamine 3000 according to the manufacturer's instructions.

### RT-qPCR

According to the instructions of Trizol RNA extraction kit (Invitrogen, USA), the transfected cells were added with Trizol reagent to extract the total RNA. The cDNA was obtained by transcribing RNA, in which the commercialized cDNA Reverse Transcription kit (Applied Biosysterms, Carlsbad, CA, USA) was used. The thermocycler conditions: 95° C for 2 min, and 35 cycles at 95° C for 20 s, and 30 s at 55° C, followed by 72° C for 60 s. All primers were designed and synthesized by Sangon Biotech (Shanghai, China). GAPDH (RAB35) and U6 (miR-185-5p) were used as an endogenous control. The expression levels were normalized using the 2^-ΔΔCt^ method.

### CCK-8 assay

We used CCK-8 assay (Sigma-Aldrich, USA) to detect A549 or H2170 cell proliferation. A549 or H2170 cells were digested with trypsin and placed in 96-well plates. The 10 μL of CCK-8 solution was added to each well at 24, 48 and 72 h, and the mixture were placed in the incubated for 2 h. A microplate reader (Molecular Devices, China) was used to measure the absorbance of each well at 450 nm.

### Transwell cell migration and invasion assay

Transwell assay was performed to measure A549 and H2170 cell invasion. Cells were seeded into the upper chamber of Transwell chambers (8.0 μm pore size; Millipore Corporation, Bedford, MA, USA) coated with Matrigel (BD Bioscience, Franklin Lakes, NJ, USA). The complete medium was added into the lower chamber. After incubation at 37° C for 48 h, cells on the upper chamber were removed with cotton swabs, while cells on the lower champer were fixed with 70% ethanol and stained with 0.1% crystal violet. The invasive cells were counted under a light microscope (Olympus, Tokyo, Japan). The cell migration assay was the same as the invasion assay step, but there was no coated Matrigel in the upper chamber in the migration assay.

### Luciferase reporter gene analysis

Potential target genes of miR-185-5p were predicted by Starbase 3.0 (http://starbase.sysu.edu.cn/), and RAB35 was screened out as the research object. The binding sites of RAB35 3’UTR were amplified and subcloned into the pmirGLO vectors (Promega) to construct RAB35 3’UTR wild-type reporter (RAB35-WT) and mutated-isoform (RAB35-MUT) encompassing mutated binding sites. The luciferase plasmids RAB35-WT and RAB35-MUT were co-transfected with miR-185-5p mimic into cells. Next, the Reporter Assay Kit (Thermo Fisher, USA) was used to detect the fluorescence intensity, which indicated the activities of the luciferase.

### Western blotting

The total protein of cells was obtained according to the reported method. The content of protein was tested by BCA kit (Solarbio, China). Then, all protein samples were denatured with SDS-PAGE loading buffer for 5 min in boiling water. Protein samples were detached on 10% SDS-PAGE and transferred to PVDF membranes (Bio-Rad, Hercules, CA, USA). The membranes were blocked using 5% skimmed milk for 3 h and then the primary antibodies were added to incubate with membranes at the 4° C overnight. The primary antibodies (Abcam) used for study were: GAPDH (1:800, EPR16769), RAB35 (1:700, ab152138), TSG101 (1:1500, ab125011), CD63 (1:1000, EPR21151), HSP70 (1:1000, EPR16892). Next, the TBST was used to wash the membranes five times, and then the HRP-conjugated goat anti-rabbit/mouse IgG (1:2000, Abcam) was added in the mixture at 25° C for 1 h. Finally, the membranes were visualized with a substrate super ECL Plus solution (Pierce, Rockford, IL, USA). The image was processed with the Image J software (Rawak Software, Carlsbad, CA, USA).

### Exosome isolation and purification

Cells (2×10^6^) were seeded in Petri dishes with RPMI containing 10% FBS. 24 h later changed to RPMI without exosomes and with 10% FBS. Cell culture supernatants were collected, and exosomes were collected by differential centrifugation: the supernatant of cell culture was collected, and the cell components and dead cells were removed by low-speed centrifugation (300 g×10 min, 2,000 g×10 min) at 4° C. The supernatant containing exosomes was retained and the cell debris was removed by high-speed centrifugation (10,000 g×70 min). The supernatant containing extracellular vesicles was retained and the exosomes were precipitated by ultracentrifugation (100,000 g×70 min). Appropriate amount of PBS was taken to resuspend the extracellular vesicle precipitation and then ultracentrifuged again (100,000 g×70 min) to eliminate contaminated proteins.

### Exosome enzyme treatment

Previously collected exosomes were hydrolyzed with RNase (2 μg/mL) for 10 min at 37° C, followed by the addition of RNase inhibitor (10U/mL). Subsequently proteinase K was added for hydrolysis at 37° C, after 60 min the temperature was adjusted to 60° C and incubated for 10 min. Then at 4° C 110,000 × g after centrifugation for 70 min, the supernatant was discarded and the resulting pellet was exosomes.

### Statistical analysis

The results were performed as mean ± SEM. All statistical analyses were exerted using the SPSS 22.0 software (Chicago, IL, USA). Student’s t-test (comparison between the two groups) and One-way ANOVA test (comparison between multiple groups) were used assessed P values. Values of *P* < 0.05 were considered statistically significant.
